# Liver-specific Gene Delivery Using Engineered Virus-Like Particles of Hepatitis E Virus

**DOI:** 10.1038/s41598-019-38533-7

**Published:** 2019-02-07

**Authors:** Eun Byul Lee, Jung-Hee Kim, Wonhee Hur, Jung Eun Choi, Sung Min Kim, Dong Jun Park, Byung-Yoon Kang, Gil Won Lee, Seung Kew Yoon

**Affiliations:** 1 The Catholic University Liver Research Center & WHO Collaborating Center of Viral Hepatitis, Department of Biomedicine & Health Sciences, The Catholic University of Korea, Seoul, 06591 Republic of Korea; 2 Present Address: am SCIENCES, C-912, SK V1 GL Metrocity, 128, Beobwonro, Songpa-gu, Seoul, 05854 Republic of Korea; 3 Present Address: 1014, A Building Gangseo-Hangang-Xi Tower 401 Yangcheon-ro, Gangseo-gu, Seoul, 157-801 Republic of Korea

## Abstract

Virus-like particles (VLPs) possess great potential for organ-specific transport of therapeutic agents due to their central cavity surrounded by viral capsid proteins and similar tropism to their original viruses. The N-terminal truncated second open reading frame (Nt-ORF2) of the hepatotropic hepatitis E virus (HEV) forms VLPs via self-assembly. In the present study, we investigated whether HEV-LPs could deliver foreign genes specifically to the liver. HEV-LPs were obtained from Nt-ORF2 expression in Huh7 cells that were transduced with recombinant baculoviruses and purified by continuous density gradient centrifugation. The purified HEV-LPs efficiently penetrated liver-derived cell lines and the liver tissues. To evaluate HEV-LPs as gene delivery tools, we encapsulated foreign plasmids in HEV-LPs with disassembly/reassembly systems. Green fluorescence was detected at higher frequency in liver-derived Huh7 cells treated with HEV-LPs bearing GFP-encoding plasmids than in control cells. Additionally, HEV-LPs bearing Bax-encoding plasmids induced apoptotic signatures in Huh7 cells. In conclusion, HEV-LPs produced in mammalian cells can encapsulate foreign genes in their central cavity and specifically transport these genes to liver-derived cells, where they are expressed. The present study could contribute to advances in liver-targeted gene therapy.

## Introduction

Chronic liver diseases, such as chronic viral hepatitis, liver cirrhosis, and liver cancer, represent severe health problems globally because of their high prevalence and the limitations of current therapies^1,2^. In particular, hepatocellular carcinoma (HCC) is one of the deadliest malignancies worldwide. Although remarkable advances have been made in the treatment of HCC, its prognosis remains poor due to accompanying progressive liver failure caused by underlying liver cirrhosis and the fact that therapeutic options are limited^3^. Therefore, it is necessary to develop novel treatment methods, such as gene therapy, for advanced HCC.

Gene therapy is considered a promising strategy with the potential to ameliorate several liver diseases by transferring therapeutic genetic materials into target cells. Some viruses have been evaluated as delivery vehicles because they possess the unique capability to deliver their genomes to the nuclei of various cells or organs^4^. Specifically, viral vectors derived from adenoviruses, retroviruses, and adeno-associated viruses have emerged as the dominant carriers of beneficial genes^5^. These viral vectors can efficiently deliver foreign genes to target cells to treat various diseases, including liver disease^6–9^. Thus, gene therapy using viral vectors is an attractive approach. However, there are some limitations in their therapeutic application: the complexity of production, limited capacity for packaging, and the possibility of insertional mutagenesis or gene inactivation. Also, repeated administration and expression over time would reduce their therapeutic efficiency^10^. Hence, many researchers have been trying to improve gene delivery systems to complement current methods.

Virus-like particles (VLPs), which are noninfectious and nonreplicating pseudo-viruses, are small particles with specific proteins derived from the outer coat of viruses. They have an inherent capacity to self-assemble, and thus they can mimic the morphology and tissue tropism of native viruses^11^. Moreover, VLPs are capable of loading not only a wide range of large molecules, such as nucleic acids^12^, peptides or proteins^13^, and other nanoparticles^14^ but also small molecules such as chemotherapeutics, fluorescent probes, and polymers^15^. Hence, it is plausible that VLPs could be used as drug carriers.

Hepatitis E virus (HEV) is a virus with selective tropism for the liver^16^. It is well-known that the major capsid protein of HEV is encoded by its second open reading frame (ORF2) and can be easily assembled to form VLPs^17^. Also, N-terminally-truncated ORF2 (Nt-ORF2), which is ORF2 protein with a deletion of 111 amino acids from the N-terminal end, can form smooth self-assembled HEV-like particles (HEV-LPs) which are popular among researchers^18,19^. Metal ions are known to play an essential role in maintaining the structure of HEV-LPs. When the metal ions are removed, the HEV-LPs structure breaks down due to the breaking of disulfide bonds, but it may be reassembled again by adding bivalent ions such as CaCl_2_^20^. Based on these properties, many researchers have tried to encapsulate various therapeutic materials in HEV-LPs. For instance, HEV-VLPs which encapsulated human immunodeficiency virus envelope (HIV env) protein were induced the immune reaction via oral administration. This significantly increased the ratio of specific IgG and IgA to HIV env in fecal extracts and sera, suggesting that HEV-VLPs could be used as tools for the delivery of foreign genes^21^.

In the present study, we attempted to deliver therapeutic agents to the liver by establishing a HEV-LPs production system using mammalian cells transduced with recombinant baculoviruses and by setting *in vitro* disassembly/reassembly systems for encapsulating foreign genes. Because these HEV-LPs could not only encapsulate genetic material via the disassembly/reassembly systems but also transport them specifically to liver-derived cells, HEV-LPs may have great potential as a liver-specific gene delivery tool.

## Results

### Generation of purified HEV-LPs in mammalian cells transduced with Bac-Nt-ORF2

**Figure 1:**
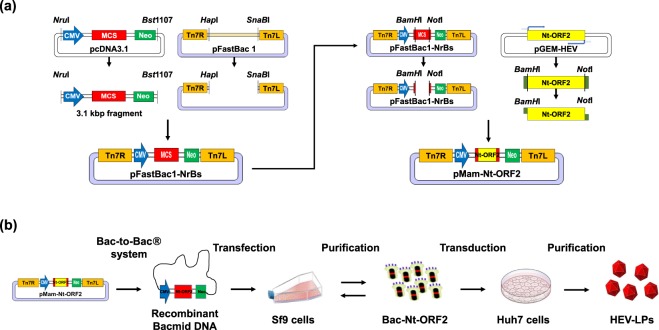
Schematic diagram of stages to create HEV-LPs. (**a**) Strategy for pMam-Nt-ORF2 construction. (**b**) Schematic diagram of HEV-LPs generation.

**Figure 2:**
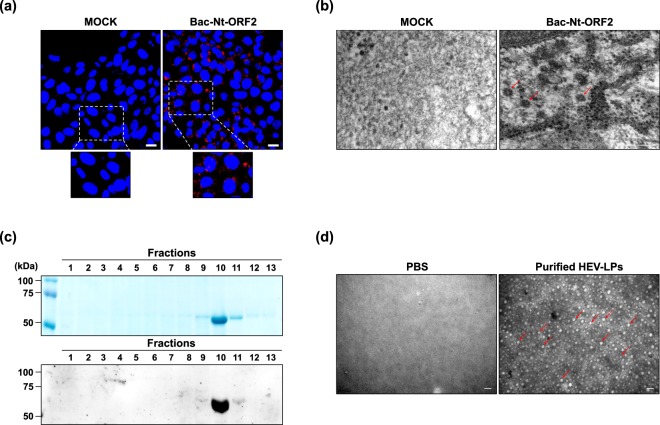
Expression of HEV Nt-ORF2 and purification of HEV-LPs in Huh7 cells. (**a**) Immunofluorescence staining of Nt-ORF2 in Huh7 cells after transduction with Bac-Nt-ORF2. Red fluorescence represents Nt-ORF2 expression. Nuclei were counterstained with DAPI. Scale bar = 20 μm. (**b**) Formation of HEV-LPs was detected by TEM in Huh7 cells after transduction with Bac-Nt-ORF2. Red arrows denote HEV-LPs in the cytosol of Huh7 cells. Scale bar = 100 nm. (**c**) Purified HEV-LPs after CsCl-density gradient centrifugation were verified by CBB staining (upper panel) and western blot assay (lower panel). (**d**) Purified HEV-LPs were observed by TEM. Red arrows denote purified HEV-LPs. Scale bar = 50 nm.

First, to produce HEV-LPs in Huh7 cells, recombinant baculoviral particles, Bac-Nt-ORF2, were generated as described in the section “Methods” (Fig. 1). Huh7 cells were transduced with Bac-Nt-ORF2, and then, the expression of Nt-ORF2 was evaluated by staining the cells with an HEV ORF2 antibody. As shown in Fig. 2a, Nt-ORF2 expression was mainly detected in Huh7 cells when transduced with Bac-Nt-ORF2. Also, the cells were observed under TEM to confirm the particle formations of HEV-LPs. The morphology of HEV-LPs was as previously reported^19^: spikes protruding from a spherical surface with dark central cavities, and their size was 23–43 nm in diameter (Fig. 2b). Next, HEV-LPs generated in Huh7 cells which had already been transduced with Bac-Nt-ORF2 were purified by sucrose-CsCl-density gradient centrifugation. After a series of centrifugations, the purified HEV-LPs were recovered from the middle fractions at approximately 1.27 g/mL^22^. After using an HEV ORF2 antibody, Coomassie Brilliant Blue (CBB) staining and western blot analysis revealed that recovered HEV-LPs were cleanly purified with no extra bands (Fig. 2c). Furthermore, the HEV-LPs structure was maintained during the purification procedure (Fig. 2d). These results indicate that HEV-LPs were successfully formed in Huh7 cells expressing Nt-ORF2 and that intact HEV-LPs could be obtained by the purification procedure.

### Intracellular penetration of purified HEV-LPs to liver-derived cells

**Figure 3:**
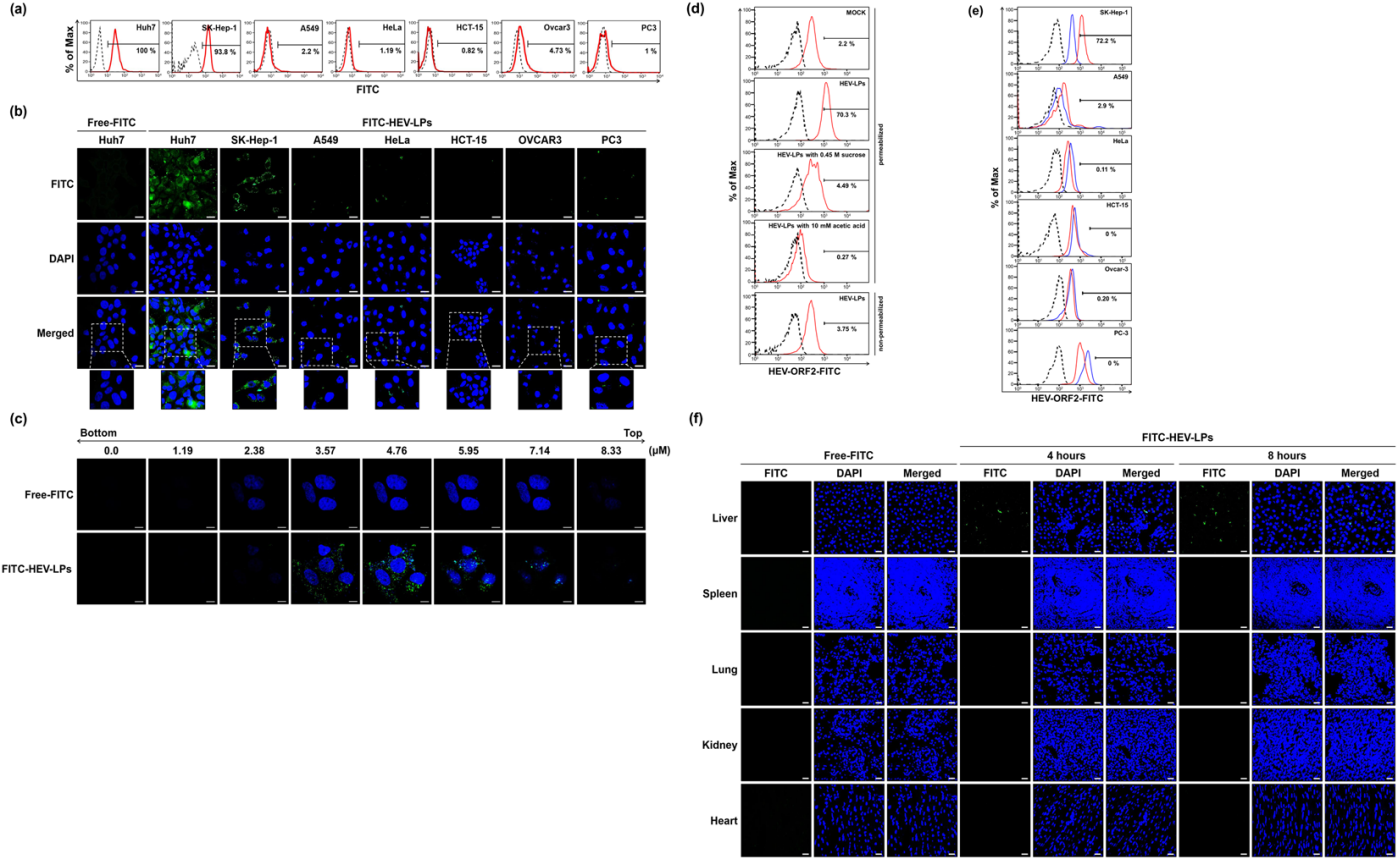
Specific penetration of HEV-LPs in liver-derived cell lines. Intracellular penetration of purified HEV-LPs in various mammalian cells was assessed by flow cytometry and confocal microscopy. (**a**) The percentage of the cells presenting green fluorescence was determined by flow cytometry after treatment with FITC-HEV-LPs. The black line indicates the fluorescence intensity of cells treated with free FITC. The red line indicates the fluorescence intensity of cells treated with FITC-HEV-LPs. (**b**) FITC-HEV-LPs were observed as green fluorescence in liver-derived cell lines by confocal microscopy. Nuclei were counterstained with DAPI. Scale bar = 20 μm. (**c**) Z-stack images were collected at 1.19 μm intervals from 0 to 8.33 μm. Green fluorescence represents FITC-HEV-LPs, Nuclei were counterstained with DAPI and red fluorescence represents cell membranes. Scale bar = 10 μm. (**d**) The percentage of HEV-LPs in Huh7 cells was determined by flow cytometry. The cells were treated with proteinase K. Huh7 cells treated with HEV-LPs with permeabilization and without permeabilization are shown separately. Huh7 cells treated with HEV-LPs were additionally treated with 0.45 M sucrose and 10 mM acetic acid. The black line indicates the second antibody control. The red line indicates the fluorescence intensity of HEV-LPs. (**e**) The percentage of cells penetrated by HEV-LPs in various cell lines was determined by flow cytometry. The cells were treated with proteinase K. The black line indicates the second antibody control. The blue line indicates the fluorescence intensity of HEV-ORF2 antibody control. The red line indicates the fluorescence intensity of HEV-LPs. (**f**) FITC fluorescence in various tissues was investigated in Wistar rats injected FITC-HEV-LPs under a confocal microscopy. The green fluorescence was represented in the section of liver which FITC-HEV-LPs injected rats regardless of sacrifice times. Nuclei were counterstained with DAPI. Scale bar = 20 μm.

To investigate whether purified HEV-LPs could specifically penetrate liver-derived cells, six different human cancer cell lines were treated with equal concentrations of fluorescein isothiocyanate (FITC)-conjugated HEV-LPs (FITC-HEV-LPs) or Free FITC to monitor the intracellular uptake of HEV-LPs. After 4 h, the intracellular penetration of FITC-HEV-LPs was assessed by flow cytometry. As shown in Fig. 3a, FITC-positive cells were detected at a higher frequency in liver-derived cell lines (Huh7 cells and SK-Hep-1 cells) than in Free FITC-treated cells. On the contrary, few FITC-positive cells were detected in cell lines derived from different organs. The percentage of FITC-fluorescence was 2.2% in A549 cells, 1.19% in HeLa cells, 4.73% in OVCAR-3 cells, 0.82% in HCT-15 cells, and 1% in PC3 cells (Fig. 3a). Subsequently, the penetration of HEV-LPs in liver-derived cell lines was confirmed by a confocal microscopy. In both Huh7 cells and SK-Hep-1 cells treated with FITC-HEV-LPs, green fluorescence in the cytoplasm was more prominent than in other cell lines (Fig. 3b). However, the liver-derived cell lines exhibited the most fluorescence. These results suggest that HEV-LPs could readily penetrate the liver-derived cell lines because of their selective tropism for liver cells.

In addition, cells treated with FITC-HEV-LPs or Free FITC were also observed by z-stacking. GFP expression was observed within cells treated with FITC-HEV-LPs. On the other hand, control experiments with Free FITC showed no fluorescence in the inner part of the z-stack images. These data were subsequently corroborated by optical sectioning of the cells at 1.19 μm intervals from top to bottom with a total section stacking of 8.33 μm (Fig. 3c). Furthermore, we analyzed the levels of intracellular HEV-ORF2 proteins in cells treated with HEV-LPs by flow cytometry. As shown in Fig. 3d, HEV-ORF2 proteins were detected inside cells treated with HEV-LPs. A previous study showed that HEV entered the cytoplasm via clathrin-mediated endocytosis^23^; hence, we evaluated whether HEV-LPs uptake was blocked by inhibitors of clathrin-mediated endocytosis (sucrose and acetic acid). Huh7 cells treated with the inhibitors showed lower levels of HEV-ORF2 proteins than those treated with HEV-LPs (Fig. 3d). Also, when HEV-LPs- treated cells were stained without permeabilization to test for the presence of HEV-LPs on the Huh7 cell surface, HEV-ORF2 proteins were not identified in the cells as expected (Fig. 3d). Moreover, HEV-ORF2 proteins were detected in liver-derived cell lines such as SK-Hep-1 whereas they were not detected in cell lines derived from different organs (Fig. 3e). Subsequently, FITC-HEV-LPs were injected into Wistar rat via the tail vein to observe *in vivo* liver-specific delivery. Wistar rat was also injected Free-FITC as a negative control. The rats were sacrificed on hour 4 and 8 and then the liver, kidney, spleen, lung, and heart of rats were extracted and harvested for imaging of the individual organs. After DAPI staining, we examined the fluorescence of the various organs under the confocal microscopy. The green fluorescence was detected in the liver tissues which were injected FITC-HEV-LPs, whereas those were rarely detected in the other organ tissues. In particular, FITC fluorescence was more observed in the liver tissues of 8 h than the liver tissues of 4 h after injection (Fig. 3f). Moreover, cell death or other pathologic changes in hematoxylin and eosin (H&E) stained tissues were not observed in various organs (Fig. S1). These results demonstrate that HEV-LPs exhibited liver tropism of the characteristic of HEV, and HEV-LPs could be dominantly penetrated liver-derived cell lines as well as the liver.

### Encapsulation of foreign plasmids in HEV-LPs

**Figure 4:**
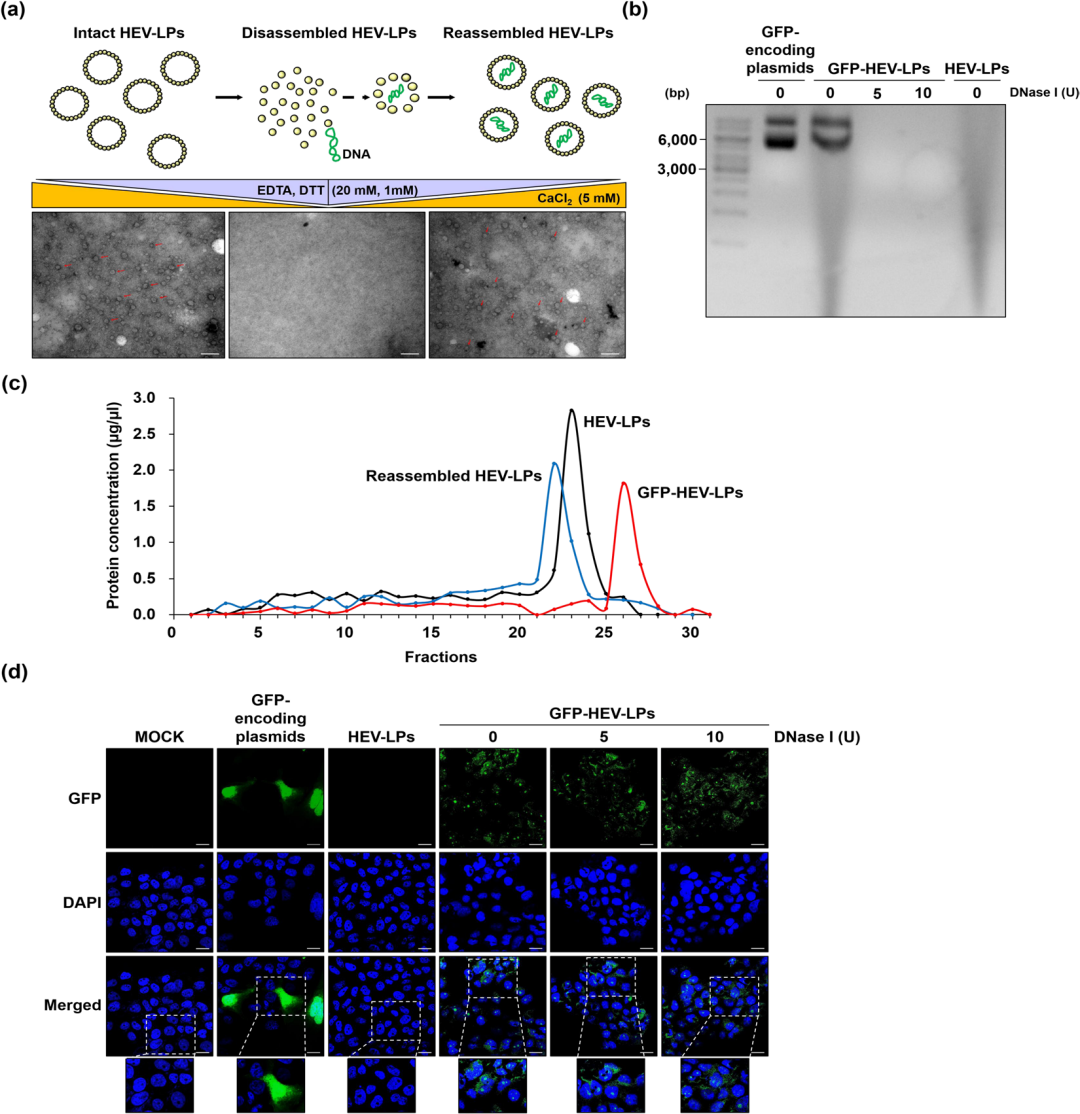
Encapsulation of GFP-encoding plasmids. (**a**) Schematic diagram of the disassembly/reassembly HEV-LPs process. Electron micrographs of HEV-LPs were obtained under different concentrations of cations. Left) Purified HEV-LPs, Middle) Disrupted HEV-LPs, Right) Reassembled HEV-LPs. Red arrows denote HEV-LPs. Scale Bar = 100 nm. (**b**) DNA gel electrophoresis of plasmids encapsulated by HEV-LPs. GFP-encoding plasmids (lane 1) and GFP-encoding plasmids within GFP-HEV-LPs (lane 2) were detected an agarose gel. GFP-encoding plasmids digested by 5 U and 10 U DNase I after heating GFP-HEV-LPs (lane 3 and 4). HEV-LPs were also co-electrophoresed to confirm the absence of DNA contamination (lane 5). (**c**) CsCl-density gradient centrifugation of various types HEV-LPs. The total protein in each fraction (400 μl) was determined by a Bradford assay. Black line indicates empty HEV-LPs. Blue line indicates disassembled and reassembled HEV-LPs. And, Red line indicates GFP-HEV-LPs. (**d**) To prove protection of encapsulated foreign genes, Huh7 cells treated with both GFP-HEV-LPs and different doses of DNase I and then were observed by a confocal microscopy. Green fluorescence indicates expression of GFP. Nuclei were counterstained with DAPI. Scale bar = 20 μm.

Because HEV-LPs could easily penetrate liver-derived cell lines, we further examined the ability of HEV-LPs to transport foreign genes into the cells. First, we established disassembly/reassembly systems to encapsulate foreign genes in HEV-LPs. It has been reported that disulfide bonds and metal ions were essential for capsid formation in HEV^20^. Hence, intact HEV-LPs were treated with chelates, such as ethylenediaminetetraacetic acid (EDTA) and dithiothreitol (DTT), to disrupt the structure of HEV-LPs. As shown in Fig. 4a, intact HEV-LPs (left) were completely disrupted (middle) by the increased concentrations of EDTA and DTT. Subsequently, HEV-LPs were reassembled when the concentration of CaCl_2_ was higher (Fig. 4a, right). Interestingly, TEM showed that morphology and size of the reassembled HEV-LPs was well-maintained following the disassembly/reassembly process. These results demonstrate that the HEV-LPs disassembly/reassembly systems could be manipulated by modifying the concentrations of calcium and chelates without changes to HEV-LPs morphology. We also used GFP-encoding plasmids to keep track of the delivery of foreign genes encapsulated in HEV-LPs. The plasmids were mixed with disrupted HEV-LPs in disassembly buffers. At higher CaCl_2_ concentrations, GFP-encoding plasmids mixed with the disrupted HEV-LPs became reassembled HEV-LPs (GFP-HEV-LPs). To identify the presence of the GFP-encoding plasmids within HEV-LPs, GFP-HEV-LPs were disrupted with SDS-PAGE loading buffers and heating. To confirm DNA mobility of the GFP-encoding plasmids released from the disrupted GFP-HEV-LPs, the plasmids were compared with naked GFP-encoding plasmids on agarose gels. The GFP-encoding plasmids released from the disrupted GFP-HEV-LPs had the same DNA mobility of the GFP-encoding plasmids (Fig. 4b, lanes 1 and 2). Then, to evaluate the characteristics of the GFP-encoding plasmids released from disrupted GFP-HEV-LPs, disrupted GFP-HEV-LPs were treated with DNase I. We found that digestion of GFP-encoding plasmids was complete with DNase I (Fig. 4b, lanes 3 and 4), and HEV-LPs displayed no DNA bands. These findings imply that there was no contaminating DNA in HEV-LPs prior to the encapsulation experiment (Fig. 4b, lane 5). We further examined encapsulation of foreign plasmids in the reassembled HEV-LPs using CsCl gradient centrifugation. As shown Fig. 4c, lighter density peaks were showed at fraction 22 and 23 which represented HEV-LPs alone and reassembled HEV-LPs, respectively. When the peak of GFP-HEV-LPs shifted to a higher density fraction 26 that was demonstrated the presence of denser HEV-LPs (Fig. 4c). Thus, HEV-LPs density was greater when encapsulated with foreign plasmids. Subsequently, to identify the protection of GFP-encoding plasmids in HEV-LPs against external factor, Huh7 cells were treated with GFP-HEV-LPs under different concentration of DNase I. The expression of GFP was observed a regardless dose of DNase I in the cells (Fig. 4d). Taken together, these results indicate that the encapsulated foreign plasmids in HEV-LPs could be manipulated using *in vitro* disassembly/reassembly systems and HEV-LPs could protect foreign plasmids in the central cavity of themselves against external factors.

### Transport of foreign gene-encoding plasmids bearing HEV-LPs to specific target cells

**Figure 5:**
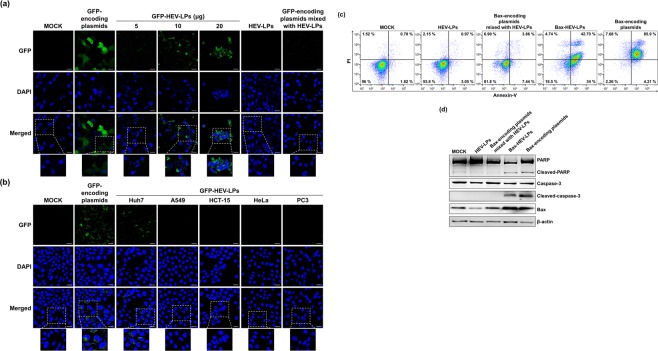
Foreign gene delivery. (**a**) Huh7 cells treated with various doses of GFP-HEV-LPs or GFP-encoding plasmids mixed HEV-LPs were observed by confocal microscopy. Green fluorescence indicates expression of GFP. Nuclei were counterstained with DAPI. Scale bar = 20 μm. (**b**) Expression of GFP-encoding plasmids was confirmed by green fluorescence in a liver-derived cell line, Huh7, by confocal microscopy. Nuclei were counterstained with DAPI. Scale bar = 20 μm. (**c**) Huh7 cells were treated with empty HEV-LPs, Bax-encoding plasmids mixed with empty HEV-LPs, or Bax-HEV-LPs, or transfected with Bax-encoding plasmids using FuGENE HD. Apoptosis of the cells was quantified by Annexin V-PI/flow cytometry. (**d**) Expression of apoptotic-related proteins, cleaved-caspase3, and cleaved-PARP after treatment with empty HEV-LPs, HEV-LPs mixed with Bax-encoding plasmids, and Bac-Nt-ORF2 was evaluated by western blot analysis. Also, Huh7 cells transfected with Bax-encoding plasmids were added as a positive control for Bax activity. β-actin was used as an internal control for loading.

To evaluate the expression of foreign genes encapsulated in HEV-LPs, Huh7 cells were treated with HEV-LPs bearing foreign gene-encoding plasmids. Huh7 cells were treated with GFP-HEV-LPs to observe the expression of GFP-encoding plasmids. We also compared the transduction efficiency of GFP-HEV-LPs to that of a commercial transfection reagent. As shown in the middle panel of Fig. 5a, green fluorescence was detected in the Huh7 cells treated with GFP-HEV-LPs; the intensity of the GFP expression was dose-dependent. To exclude the possibility that naked GFP-encoding plasmids would penetrate the cells, the cells were treated with GFP-encoding plasmids mixed with empty HEV-LPs. As shown in Fig. 5a, no green fluorescence was detected within the cells treated with GFP-encoding plasmids mixed with empty HEV-LPs. These results show that HEV-LPs can penetrate cells and that foreign genes released from HEV-LPs were expressed in the treated cells. Next, to examine the liver-specific delivery function of HEV-LPs bearing foreign genes, five diverse cell lines were treated with equal concentrations of GFP-HEV-LPs. As with the results shown in Fig. 4b, green fluorescence was detected at a higher frequency in the liver-derived cell lines, particularly in Huh7 cells (Fig. 5b). On the other hand, green fluorescence was hardly detected at all in the five other cell lines.

Although we observed the permeability of cells to HEV-LPs, it is unclear whether HEV-LPs affect cell function. To resolve this question, we treated Huh7 cells with Bax-HEV-LPs bearing Bax-encoding plasmids. Bax is a pro-apoptotic protein and its overexpression can lead to apoptotic cell death^24^. When Bax-encoding plasmids were transfected with FuGENE HD, ectopic expression of Bax in transfected cells caused significant increases in both the early and late apoptosis. However, apoptosis was not observed in empty HEV-LPs and Bax-encoding plasmids mixed with HEV-LPs. Interestingly, treating Huh7 cells with Bax-HEV-LPs also increased both early and the late apoptosis and left apoptotic signatures (Fig. 5c). Also, expression of cleaved caspase-3 and PARP were detected by western blot analysis, indicating that of the apoptotic signaling pathway was induced (Fig. 5d). Taken together, these results suggest that foreign genes encapsulated in HEV-LPs maintain their original functions.

## Discussion

In the present study, we determined that HEV-LPs can function as liver-specific gene delivery vehicles. The properties of HEV-LPs were inherited from HEV, which exhibits liver tropism. We attempted to safely and efficiently produce HEV-LPs in mammalian cells to determine their function as liver-specific gene delivery tools and then verified their function by observing intracellular penetration in liver-derived cell lines along with FITC-HEV-LPs. Furthermore, FITC-HEV-LPs were dominantly delivered to the liver of rats via intravenous injection. Additionally, we established disassembly/reassembly systems to encapsulate foreign genetic material, such as GFP- or Bax-encoding plasmids, into the central cavity of the HEV-LPs. Interestingly, in our systems, the encapsulated foreign genes were readily delivered and overexpressed in liver-derived cells. Also, the overexpressed foreign genes carried out their respective functions, such as fluorescence or induction of apoptosis. These results show that HEV-LPs may be useful as liver-specific gene delivery tools.

We also established an expression system for Nt-ORF2 using recombinant baculoviruses containing a CMV promoter-derived gene in mammalian cells. Previous studies sought to express foreign genes in recombinant baculoviruses under the control of mammalian expression cassettes^25–27^. Recombinant baculoviruses function as gene delivery agents in mammalian cells. Their advantages include 1) the inability to replicate in mammalian cells, 2) apparent lack of cytotoxicity, 3) the capacity to sustain many inserted foreign DNAs, and 4) safer features than mammalian virus-based transduction systems^28^. In a previous study, cells of human HCC cell line, Huh7 could be infected with recombinant baculoviruses and were able to express the encoding genes driven by a CMV-promoter contained in the recombinant baculovirus. The expression levels of the encoding genes were confirmed in an order of magnitude higher than in HepG2 cells^26^. For these reasons, in the present study, we designed a recombinant baculovirus (Bac-Nt-ORF2) to easily produce HEV-LPs in mammalian Huh7 cells. We established HEV-LPs generation systems and obtained HEV-LPs produced in Huh7 cells that were transduced with Bac-Nt-ORF2.

Recently, many researchers have endeavored to utilize VLPs as biomaterial transporters^11^. For instance, capsid proteins of *Macrobrachium rosenbergii* nodavirus (MrNv)^20^ and human papillomavirus (HPV)-16^29^ are able to self-assemble so as to form VLPs and encapsulate foreign genes the VLPs central cavity. Previous studies have verified that self-assembling VLPs can encapsulate foreign genes via disassembly/reassembly systems^20,29,30^. Specifically, the N-terminal truncated form of HEV-ORF2 (Nt-ORF2) of HEV capsid structural proteins is able to self-assemble. Furthermore, studies have described how HEV-LPs could encapsulate unrelated plasmid constructs^21,31,32^. In the present study, we identified that foreign genes encapsulated in HEV-LPs exhibited liver tropism due to the properties of HEV-LPs. It is noteworthy that the original function of the foreign genes encapsulated in HEV-LPs is retained even after they enter the cells. Therefore, HEV-LPs have therapeutic potential for chronic liver disease by delivering genes to the liver.

Gene delivery to liver-derived cells has already been explored using HBV- and HEV-like particles^33,34^. In those experiments, foreign molecules were displayed on the particle surface by recombinant expression vectors where the viral capsid protein and foreign genes were encoded. When primary hepatocytes or Huh7 cells were transfected with the recombinant vector, VLPs displaying foreign molecules on their surfaces were produced. The drawback of this system is that it is inconvenient to deliver foreign molecules expressed on the VLPs surface because, in gene therapy, recombinant vectors should be redesigned when the foreign molecules change. However, as our results showed, *in vitro* HEV-LPs disassembly/reassembly systems can encapsulate various therapeutic genes without any changes in the HEV-LPs structures. Furthermore, utilization of HBV-like particles as gene-delivery tools in clinical settings can be difficult because of both a lack of small animal models that can be infected by HBV and the absence of established encapsulation methods for disassembly/reassembly systems. However, HEV could replicate in a small animal model, such as the Wistar rat^35^. It is a huge advantage that small animals are available for HEV infection when experimenting with HEV-LPs whether in nonclinical or clinical trials.

In summary, we produced HEV-LPs in mammalian cells via recombinant baculoviruses transduction and used them to transport foreign genes to liver-derived cell lines. Based on the capabilities of HEV-LPs, we established *in vitro* disassembly/reassembly systems for foreign gene encapsulation in HEV-LPs. Finally, we found that functional genes which were encapsulated in HEV-LPs were expressed and activated in liver-derived cells. Our study could contribute to advances in liver-targeted gene therapy for incurable liver diseases.

## Methods

### Cell culture

Sf9 cells derived from *Spodoptera frugiperda* were grown in SF900-II (Gibco, Carlsbad, CA, USA) at 27 °C. Huh7 cells and SK-Hep-1 cells derived from the liver, A549 cells derived from the lung, Ovcar3 cells derived from the ovary, and HeLa cells derived from the cervix were grown in DMEM (Gibco). HCT-15 cells derived from the colon, and PC3 cells derived from prostate were grown in RPMI 1640 (Gibco). Mammalian cells were supplemented with 10% fetal bovine serum, 1% penicillin/streptomycin and were incubated in a humidified incubator at 37 °C with 5% CO_2_.

### Plasmids

To deliver and express HEV Nt-ORF2 in Huh7 cells, we generated a recombinant baculovirus, Bac-Nt-ORF2, carrying mammalian expression cassettes to direct gene expression in mammalian cells. pFastBac1-NrBs were constructed using pFastBac1 vector (Gibco) and pcDNA3.1 vector (Invitrogen, Carlsbad, CA, USA). pFastBac1 was digested with *Hpa*I and *Sna*BI to remove the baculovirus polyhedrin gene promoter sequences. pcDNA3.1 was digested with *Nru*I and *Bst*1170 to generate a fragment containing the CMV promoter, a multiple cloning site, and a polyadenylation signal, followed by the SV40 promoter-neomycin phosphotransferase II expression cassette (3.1 kbp fragment). Both digested fragments from pFastBac1 and pcDNA3.1 were ligated to construct pFasBac1-NrBs in frame (Fig. 1a, left)^25^. Subsequently, to create pMam-Nt-ORF2, recombinant baculovirus vector, Nt-ORF2 genes were synthesized by Bioneer Corporation (Seoul, Republic of Korea) and amplified using PCR with Nt-ORF2-specific primers (HEV-LPs-F; 5′-AAGGATCCATGGCGGTCGCTCCAGCCCATGACACCCC GCCAGT-3′ and HEV-LPs-R; 5′-AAGCGGCCGCCTATGCTAGCGCAGAGTGGGGG GCTAAAA-3′). The PCR products were cut with *Bam*HI and *Not*I and then inserted into pFastBac1-NrBs in frame (Fig. 1a, right). pMam-Nt-ORF2 was composed of the Tn7 regions of pFastBac1 for the homologous recombinant baculovirus and the Nt-ORF2 gene, which were controlled by the CMV promoter of pcDNA 3.1. GFP-encoding plasmids (pEGFP-C1) were purchased from Clontech Laboratories (Mountain View, CA, USA).

### Generation of recombinant baculovirus

The recombinant baculovirus vector (pMam-Nt-ORF2) was transformed into DH10Bac^TM^ competent cells (Invitrogen) to generate recombinant Bacmid DNA by Bac-to-Bac^®^ Baculovirus Expression System (Gibco) according to the manufacturer’s recommendations. The recombinant Bacmid DNA was transfected into Sf9 cells by using CellfectinII reagent (Invitrogen) to generate recombinant baculoviral particles (Bac-Nt-ORF2)^36^. Bac-Nt-ORF2 was recovered from the culture media. The infectious titers of Bac-Nt-ORF2 were determined by Baculovirus titration kits (Clontech Laboratories) according to the manufacturer’s recommendations.

### Purification of HEV-LPs

After generation of Bac-Nt-ORF2 in Sf9 cells, Huh7 cells were transduced with Bac-Nt-ORF2 to express Nt-ORF2, resulting in HEV-LPs formation. These steps are presented schematically in Fig. 1b. After 3 × 10^6^ Huh7 cells were seeded, the cells were transduced with Bac-Nt-ORF2 at a multiplicity of infection of 5 in serum-free media. After 12 h, the cell culture media was replaced with complete DMEM. Three days later, the cells were harvested and lysed by several freeze-thaw cycles. The intact cells, cell debris, and progeny baculovirus were removed by centrifugation at 10,000 × g for 30 min. The supernatant was further centrifuged at 130,000 × g for 3.5 h in a SW41Ti rotor (Beckman Coulter, Brea, CA, USA). The resulting pellets were suspended and incubated in PBS at 4 °C overnight. For sucrose-gradient centrifugation, the samples were placed on top of a 10–50% (w/w) sucrose gradient and centrifuged at 130,000 × g for 3.5 h in a SW41Ti rotor and recovered from the middle fractions of the sucrose density gradient, further purified by CsCl-density gradient centrifugation at 130,000 × g for 22 h in a SW41Ti rotor. HEV-LPs were recovered from the middle fractions of the CsCl-density gradient. Purified HEV-LPs were confirmed by SDS-PAGE followed by CBB staining or western blot analysis using an antibody against HEV-ORF2 (Abcam, Cambridge, UK). Finally, the collected HEV-LPs were dialyzed against PBS^37–39^.

### Transmission electron microscope (TEM)

Huh7 cells transduced with Bac-Nt-ORF2 were fixed using 4% paraformaldehyde and 2.5% glutaraldehyde in 0.1 M phosphate buffer (pH 7.2) at 4 °C overnight. And, to observe the morphology of VLPs, HEV-LPs were loaded on glow-discharged, carbon-coated copper grids and were stained with 2% uranyl acetate. The samples were then processed for TEM as described previously^40^.

### Conjugating HEV-LPs with fluorescein isothiocyanate (FITC)

To trace HEV-LPs after transduction into cells, purified HEV-LPs were conjugated with FITC (Thermo Fisher Scientific, Rockford, IL, USA) according to the manufacturer’s instructions. Briefly, 350 μg HEV-LPs (5.6 μM) (MW: 55,500 Da) was dissolved in 2 mL PBS, and 10 mg FITC was dissolved in 1 mL dimethylformamide (Thermo Fisher Scientific) (25 mM). The solution was mixed at a molar ratio of 1:100 (HEV-LPs: FITC = 5.6 μM: 560 μM) and incubated for 2 h at RT in the dark. FITC-conjugated HEV-LPs were dialyzed three times using 10 kDa cutoff dialysis tubing (Thermo Fisher Scientific) in 3 L of PBS to remove unconjugated FITC. In theory, this process would stop once the concentration of the FITC-HEV-LPs and PBS on either side of the membrane of dialysis tubing equalized. We used free FITC in PBS as a negative control. The amount of FITC-HEV-LPs was determined using a Bradford protein assay kit (Bio-Rad Laboratories, Hercules, CA, USA).

### Intracellular penetration of FITC-HEV-LPs

Six cell lines were treated with FITC-conjugated HEV-LPs or Free FITC. Following 4 h, the cells were fixed with 4% paraformaldehyde for 10 min at RT. After washing three times with PBS, the cells were stained with DAPI (1 μg/mL) (Sigma-Aldrich, St. Louis, MO, USA) for nuclear staining. After staining, intracellular penetration of FITC-HEV-LPs was observed under confocal microscope (CarlZiess, Oberkochen, Germany). Penetration was also evaluated by flow cytometry (FACSCanto II; BD biosciences, Franklin Lakes, NJ, USA). The data were analyzed with FlowJo software (Tree Star, San Carlos, CA, USA).

### Intracellular staining for HEV-ORF2 proteins

Six cell lines were seeded in 60 mm culture dishes. Next day, the cells media was changed to serum-free DMEM and then treated with HEV-LPs. After 4 h of treatment with HEV-LPs, all cells were harvested by 0.25% Trypsin-EDTA (Gibco) and then incubated with proteinase K (5 μg/mL) for 10 min at 37 °C to remove HEV-LPs attached to the cell surface. The cells were fixed and permeabilized using Intracellular Fixation and Permeabilization Buffer set (Thermo Fisher Scientific) following the manufacturer’s instructions, then stained using HEV-ORF2 antibody and Alexa Fluor 488-conjugated anti-rabbit antibody (Invitrogen). To confirm intracellular penetration of HEV-LPs, the levels of intracellular ORF2 were compared with that in cells treated with 0.45 M hypertonic sucrose or 10 mM acetic acid for 30 min at 37 °C before the addition of HEV-LPs. Intracellular penetration of HEV-LPs was evaluated by flow cytometry, and the data were analyzed with FlowJo software.

### Animal and Injection of FITC-HEV-LPs

All animal experiments were performed in accordance with institutional guidelines and were approved by the Institutional Animal Care and Use Committee of The Catholic University of Korea. Four-week-old Wistar rats (Central Lab. Animal Inc., Seoul, Korea) were housed in the animal facility for least 2 weeks before starting the experiments. To investigate liver-specific delivery of FITC-HEV-LPs, rats were received a single intravenous injection of Free-FITC as a control and FITC-HEV-LPs dose of 15 mpk (mg/kg). After 1 h or 8 h, various tissues containing liver, spleen, kidney, lung, and heart were removed aseptically and embedded in OCT compound (Leica Biosystems, Chicago, IL, USA). Tissues were sectioned at 10 μm using cryostat, mounted on glass slides. After drying, the fixed sections were stained with DAPI (1 μg/mL) to detect the fluorescent signal emitted from FITC. The sections were routinely stained with hematoxylin and eosin (H&E) and examined by light microscopy.

### Disruption of HEV-LPs and encapsulation of plasmids

Disassembly and reassembly of HEV-LPs were performed according to previously described procedures^29,31^. To disrupt the structure of HEV-LPs, purified HEV-LPs were incubated in a disassembly buffer containing 50 mM Tris-HCl buffer (pH 7.5), 150 mM NaCl, 20 mM EDTA, and 1 mM DTT at RT for 1 h. The disrupted HEV-LPs were refolded by increasing the concentration of CaCl_2_ to a final concentration of 5 mM for 1 h. For encapsulating the plasmids, GFP-encoding plasmids were slowly added to the disassembled HEV-LPs in the disassembly buffer. The disrupted HEV-LPs were refolded with GFP-encoding plasmids by increasing the concentration of CaCl_2._ Consequently, we incubated the foreign plasmids with HEV-LPs at a one-to-five ratio. After encapsulating, HEV-LPs complexes were treated with DNase I to degrade the unpackaged DNA. To verify the existence of GFP-encoding plasmids in the refolded HEV-LPs, HEV-LPs bearing GFP-encoding plasmids (GFP-HEV-LPs) were heated at 95 °C to disrupt the HEV-LPs and treated with DNase I (37 °C, 1 h) to degrade GFP-encoding plasmids released from the disrupted HEV-LPs^20^. Moreover, GFP-encoding plasmids encapsulated in the HEV-LPs was first incubated in the SDS-PAGE loading dye and heated at 95 °C for 5 min to break apart all of the tightly assembled HEV-LPs. The existence of GFP-encoding plasmids was confirmed on 1% agarose gels. The nucleic acids were visualized under UV light using Gel-Doc CQ systems (Bio-Rad Laboratories). Encapsulation of Bax-encoding plasmids into HEV-LPs applied the methods described above.

### Validation of encapsulated foreign plasmids in HEV-LPs

To identify existence of foreign plasmids inside HEV-LPs, GFP-HEV-LPs encapsulated by the disassembly/reassembly system were placed on top of a CsCl-density gradient and centrifuged at 130,000 × g for 22 h in a SW41Ti rotor. The concentration of each fraction (400 μl) was determined using a Bradford assay kit^41^. In addition, to prove protection of foreign plasmids in the central cavity of HEV-LPs against external factors, Huh7 cells were treated with both GFP-HEV-LPs and 5 U, 10 U of DNase I in serum-free medium. After 4 h the cells culture media was replaced with complete DMEM and then the cells were incubated for 48 h at 37 °C. The fluorescence of GFP was observed using the confocal microscopy. As positive controls, Huh7 cells were treated with GFP-HEV-LPs without DNase I and were transfected with GFP-encoding plasmids.

### Cell-specific delivery and expression of GFP-HEV-LPs

Huh7 cells were treated with dose-dependently GFP-HEV-LPs in serum-free medium, after 4 h the cell culture media was replaced with complete DMEM. Cells were treated with empty HEV-LPs or HEV-LPs mixed with naked GFP-encoding plasmids as negative controls. After 48 h, the expression of GFP-encoding plasmids was observed under confocal microscope. To verify cell-specific delivery of GFP-HEV-LPs, the six cell lines treated with 20 μg GFP-HEV-LPs and processed as described above. Huh7 cells were transfected with GFP-encoding plasmids using FuGENE HD (Promega, Madison, WI, USA) as a positive control.

### Cell apoptosis assay

Huh7 cells were treated with Bax-HEV-LPs in serum-free medium, after 4 h the cell culture media was replaced with complete DMEM. Cells were treated with empty HEV-LPs or HEV-LPs with naked Bax-encoding plasmids were used as negative controls. After 48 h, apoptosis was detected by Annexin V/propidium iodide (PI) staining (BD biosciences) according to the manufacturer’s instructions, then the data were analyzed using FlowJo software. Cleaved-caspase3 (Cell Signaling Technologies, Danvers, MA, USA) and PARP (BD biosciences) were assessed by western blot analysis. The analysis was performed as previously described^3^. The density of each band was analyzed using Multi Gauge V3.0 (Fujifilm, Tokyo, Japan). To confirm the cellular changes resulting from Bax overexpression, cells transiently transfected with Bax-encoding plasmids using FuGENE HD were used as a positive control.

## Supplementary information

sup 1.
