# Supplementary material for: Liver-specific Gene Delivery Using Engineered Virus-Like Particles of Hepatitis E Virus
Source: Sci Rep. 2019 Feb 7;9:1616. doi: 10.1038/s41598-019-38533-7 (PMC6367430; doi:10.1038/s41598-019-38533-7)
Supplement: Supplementary file 1 — sup 1. [file 41598_2019_38533_MOESM1_ESM.docx]

**SREP-18-35235A Supplementary Information**

**Liver-specific Gene Delivery Using Engineered Virus-Like Particles of Hepatitis E Virus**

Eun Byul Lee, Jung-Hee Kim, Wonhee Hur, Jung Eun Choi, Sung Min Kim, Dong Jun Park, Byung-Yoon Kang, Gil Won Lee and Seung Kew Yoon*****

The Catholic University Liver Research Center & WHO Collaborating Center of Viral Hepatitis, Department of Biomedicine & Health Sciences, The Catholic University of Korea, Seoul, 06591, Republic of Korea

**Supplementary Figure 1**


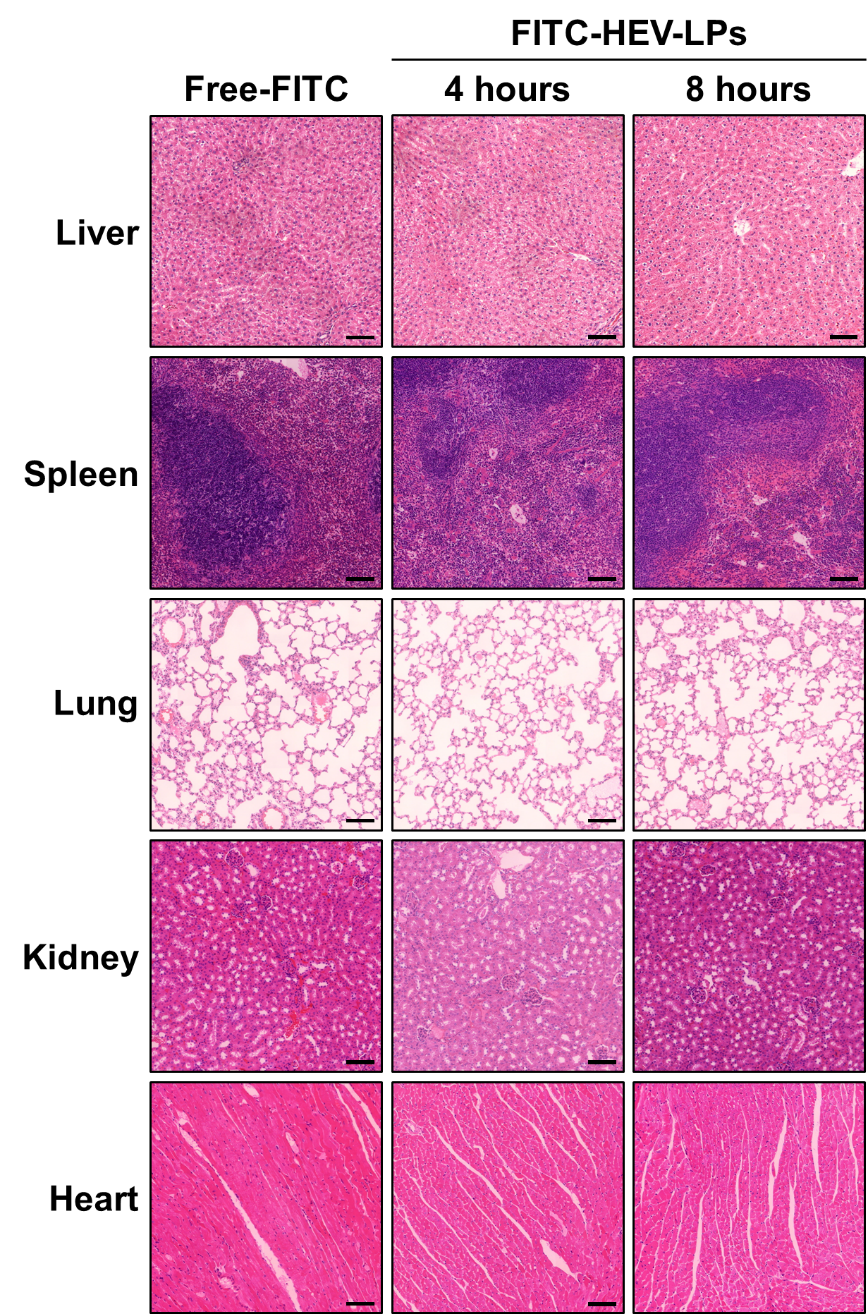


**Supplementary Figure 1. Toxicity and immunogenicity analysis of from rats after treated with Free-FTIC or FITC-HEV-LPs by H&E staining. The scale bar represents 100 μm.**
